# Metabolic network-based predictions of toxicant-induced metabolite changes in the laboratory rat

**DOI:** 10.1038/s41598-018-30149-7

**Published:** 2018-08-03

**Authors:** Venkat R. Pannala, Martha L. Wall, Shanea K. Estes, Irina Trenary, Tracy P. O’Brien, Richard L. Printz, Kalyan C. Vinnakota, Jaques Reifman, Masakazu Shiota, Jamey D. Young, Anders Wallqvist

**Affiliations:** 1 Department of Defense Biotechnology High Performance Computing Software Applications Institute, Telemedicine and Advanced Technology Research Center, U.S. Army Medical Research and Materiel Command, Fort Detrick, MD 21702 USA; 2 Department of Molecular Physiology and Biophysics, Vanderbilt University School of Medicine, Nashville, TN 37232 USA; 3 Department of Chemical and Biomolecular Engineering, Vanderbilt University School of Engineering, Nashville, TN 37232 USA

## Abstract

In order to provide timely treatment for organ damage initiated by therapeutic drugs or exposure to environmental toxicants, we first need to identify markers that provide an early diagnosis of potential adverse effects before permanent damage occurs. Specifically, the liver, as a primary organ prone to toxicants-induced injuries, lacks diagnostic markers that are specific and sensitive to the early onset of injury. Here, to identify plasma metabolites as markers of early toxicant-induced injury, we used a constraint-based modeling approach with a genome-scale network reconstruction of rat liver metabolism to incorporate perturbations of gene expression induced by acetaminophen, a known hepatotoxicant. A comparison of the model results against the global metabolic profiling data revealed that our approach satisfactorily predicted altered plasma metabolite levels as early as 5 h after exposure to 2 g/kg of acetaminophen, and that 10 h after treatment the predictions significantly improved when we integrated measured central carbon fluxes. Our approach is solely driven by gene expression and physiological boundary conditions, and does not rely on any toxicant-specific model component. As such, it provides a mechanistic model that serves as a first step in identifying a list of putative plasma metabolites that could change due to toxicant-induced perturbations.

## Introduction

Adverse effects associated with acute or chronic exposure to therapeutic drugs and toxic environmental chemicals pose serious human health concerns, including long-term debilitation, permanent organ damage, and even death. Among internal organs, the liver is the first to encounter ingested or absorbed chemicals because of its active role in the metabolism and clearance of toxicants. For example, overexposure to acetaminophen (N-acetyl-p-aminophenol, APAP), a widely used over-the-counter analgesic and antipyretic drug, is a leading cause of acute liver failure and one of the major reasons for liver transplantation in the United States (U.S.)^1^. According to the 2012 U.S. Military Health System report^2^, the prevalence of APAP overdose is higher among military personnel than among the general populace, and higher among those aged below 45 years. Furthermore, drug-induced liver injury is a major challenge for developing new drugs because it is often the primary reason that a drug is withdrawn from the market^3^. Identification of injury-specific markers would allow for an early diagnosis and, potentially, the deployment of a more effective early treatment to mitigate injury progression.

For most toxicants, the biological mechanisms that underlie the occurrence of liver injury and its transition to severe liver damage remain unknown. This limits our ability to detect early toxicant-induced changes, which, if not countered in a timely manner, can lead to irreversible alterations and functional degradation of the liver. Currently used markers for diagnosing liver injury, such as serum levels of alanine transaminase (ALT) and aspartate aminotransferase (AST), are not ideal for several reasons. For example, because these enzymes are ubiquitously expressed at similar levels in multiple organs, interpreting any change in their serum levels in response to toxicant exposure can be complicated^4–6^. Furthermore, because ALT and AST are secreted after cell death and, thus, reflect events that occur late in the cell-injury process, these enzymes may not be specific for identifying early-stage injuries that can more readily be treated.

The development of methods to screen for robust diagnostic markers characteristic of the early injury process remains a challenge. The emergence of multiple genome-scale and high-throughput technologies over the past decade has made it possible to measure multiple whole-cell physiological readouts of mRNA, proteins, microRNA, metabolites, protein-DNA binding, protein-protein interactions, and other molecules or molecular processes. However, it remains a daunting task to integrate these data into a comprehensive, mechanistic framework that clarifies how to characterize the state of a cell, tissue, or organism in terms of underlying biological processes. Metabolomics is a rapidly evolving field that can provide novel insights into toxicant-induced hepatotoxicity, mechanisms of disease pathogenesis, and markers for diagnosis and prognosis^7–9^. Here, we leverage metabolomic, fluxomic, and transcriptomic measurements from *in vivo* studies as a means to generate mechanism-based predictions of metabolite profiles, using a metabolic network modeling approach that is biologically constrained. Our study examines canonical cellular metabolism, i.e., endogenous metabolism excluding xenobiotic metabolism, to identify a sensitive and specific response to perturbations in cell physiology induced by exposure to a chemical toxicant. The goal of these studies is to provide a computational/experimental framework that can identify molecular processes specifically perturbed in a disease state and, hence, proteins and metabolites whose circulating levels in biofluids systematically differ between a disease group and a control group, and to propose these as diagnostic and prognostic markers^10–15^.

Cellular metabolism can be modeled by genome-scale network reconstructions (GENREs), which are mathematical representations of interconnected metabolic pathways that consider stoichiometric and thermodynamic constraints. These models capture metabolic phenotypes under diverse physiological and genetic conditions^16,17^, and provide a framework for elucidating genotype-to-phenotype relationships^18–22^. Several studies have used GENREs to identify metabolites as markers for different disease states. For example, a human GENRE was used to predict metabolite markers for inborn errors of metabolism^23^, and a multi-tissue mouse GENRE was used to identify high levels of branched-chain amino acids and free fatty acids in the plasma of type 2 diabetes subjects by integrating gene expression data^24^. Recently, a reconciled rat and human GENRE was developed and used to study comparative toxicogenomics and metabolite marker predictions for the effect of different drugs on liver cells in culture^16^.

In studies of GENREs that integrate transcriptomic data to discover drug targets and identify metabolite markers, the primary data used are mRNA expression profiles, as measured by microarrays or RNA sequencing (RNA-seq), and levels of small metabolites, as quantified by chromatographic separation methods in combination with mass spectrometry^7–9,25^. These studies implement different algorithmic approaches to link the different data types^23,26^. In particular, GENREs that incorporate genotype-to-phenotype relationships through gene-protein-reaction (GPR) rules provide one means for integrating transcriptomic data to identify differences in plasma metabolite levels between control and toxicant-treated conditions.

In the current study, we used male Sprague Dawley rats and acetaminophen (APAP) as the exemplar toxicant to generate *in vivo* transcriptomic and metabolomic data, as well as ^2^H/^13^C metabolic flux analysis to measure central carbon metabolism. We integrated these data into the recently developed rat GENRE (*iRno*) using a novel algorithm called Transcriptionally Inferred Metabolic Biomarker Response (TIMBR)^16^, and investigated the metabolic differences in plasma between control and APAP-treated conditions. We then characterized the efficacy of the rat GENRE in predicting the metabolites as markers of APAP-induced liver injury and identified the underlying pathways perturbed by APAP treatment based on changes in gene expression. Using these highly perturbed pathways and the metabolites within them, we identified a criterion to further classify the predicted metabolites as the most likely to be associated with the gene expression changes, and hence, selected as list of potential metabolites for further investigation. These predictions of the model may be used for further validation by targeted metabolomic analysis. More generally, the developed modeling strategy has the potential to identify a set of robustly changing metabolites as injury markers, using publicly available transcriptomic data from liver toxicity databases, and may be used to identify canonical metabolites as early markers of injury progression for other toxicant-induced organ injuries. Overall, the model developed here provides a framework for inter-relating multiple sources of omics-level data and generating a list of putative metabolites associated with toxicant-induced organ injuries based on observed changes of gene expression *in vivo* in specific tissues.

## Results

### Experimental design to monitor early physiological changes in the liver

**Figure 1:**
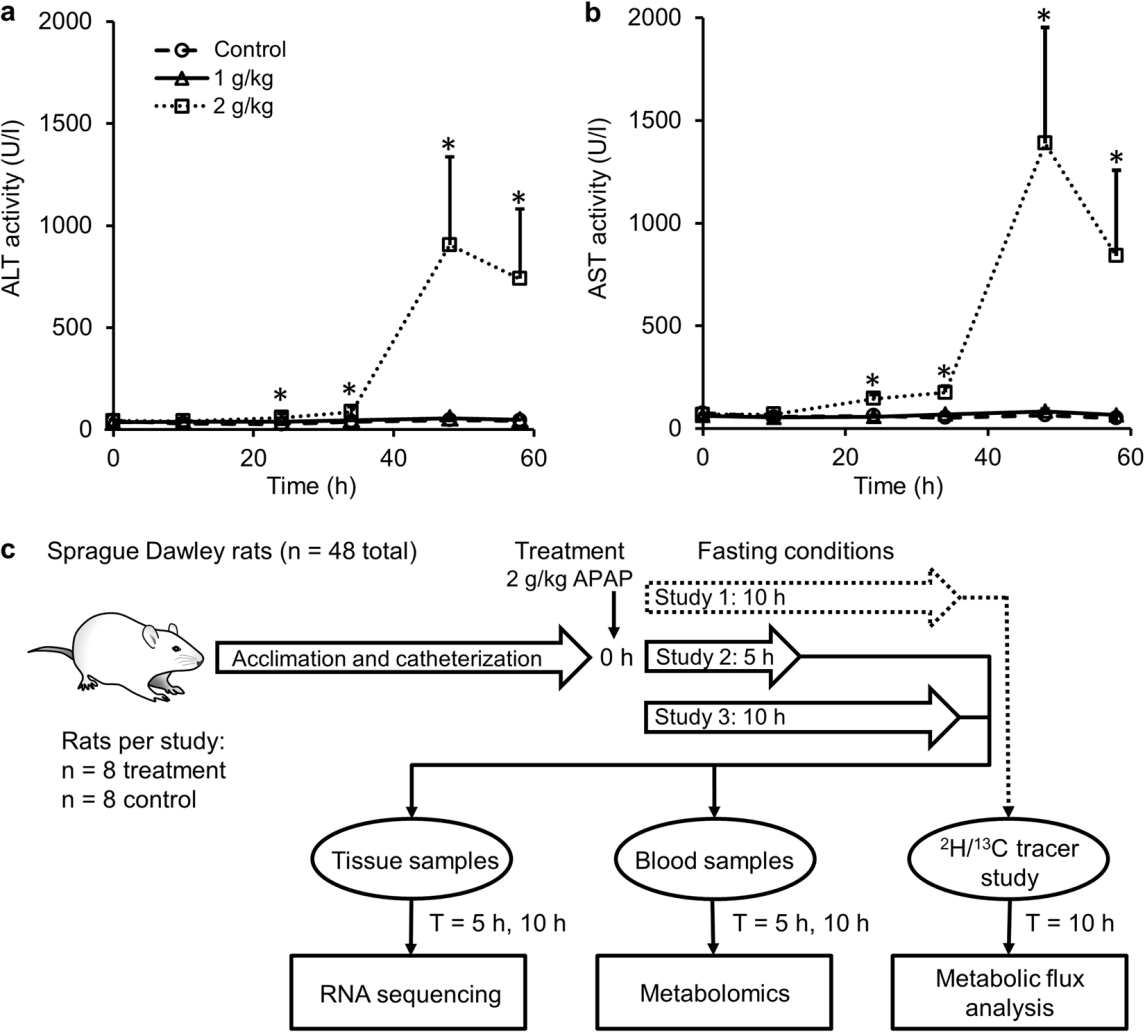
Experimental design to measure early perturbations in rat liver metabolism. Preliminary studies using clinical chemistry markers to determine acetaminophen (APAP) dose and duration. Alterations in ALT (**a**) and AST activity levels (**b**) for control (dashed line with circles, n = 6), 1 g/kg APAP (solid line with triangles, n = 6), and 2 g/kg APAP (dotted line with squares, n = 7, ^*^p < 0.05). (**c**) Schematic showing the design of the study, using Sprague Dawley rats exposed to a single dose of 2 g/kg of APAP under fasting conditions. In Study 1, rats were administered APAP and observed for 10 h (n = 8) together with control animals (n = 8), after which they were infused with ^2^H/^13^C labeling to obtain flux measurements using metabolic flux analysis. In Studies 2 and 3, rats were given APAP and observed for 5 h (n = 8) and 10 h (n = 8), respectively, with the corresponding control groups treated with vehicle (n = 8 each). Samples of blood and liver tissue were collected at 5 h or 10 h after APAP exposure and subjected to global metabolic profiling analysis or RNA sequencing, respectively (See Materials and Methods for further details).

To identify an optimal APAP dose that initially causes non-symptomatic perturbations in liver physiology before inducing extensive liver injury, we varied the APAP dose and examined the time course of injury evolution by measuring plasma levels of ALT (Fig. 1a) and AST (Fig. 1b). For animals treated with either a low (1 g/kg) or high dose (2 g/kg) of APAP, ALT and AST levels were unaltered compared to vehicle-treated controls for up to 10 h. After 10 h, whereas ALT and AST levels remained unchanged for animals given the low-dose treatment, they significantly increased for those given the high-dose treatment and reached peak levels at around 48 h before starting to decline. Subsequent histopathological analysis of liver tissue samples collected after 58 h confirmed the presence of significant liver injury for animals treated with the high dose. These results indicate that high-dose (2 g/kg) APAP treatment causes no marked elevation of ALT and AST before 10 h, but induces liver injury within 48 h after treatment.

To capture the early perturbations in liver physiology, we subjected rats to the single high-dose (2 g/kg) treatment and monitored one group of rats for 5 h and the other for 10 h, before sacrificing them to collect samples to investigate changes of global gene expression in liver tissue and blood metabolite profiles induced by APAP under fasting conditions (Fig. 1c). Additionally, we performed ^2^H/^13^C tracer studies under the same single high dose (2 g/kg) treatment, and collected samples at the 10-h time point to identify the major fluxes within the glucose production pathway of central carbon metabolism under fasting conditions (Fig. 1c; see Methods Section for details).

### APAP-induced gene expression changes in the liver

**Figure 2:**
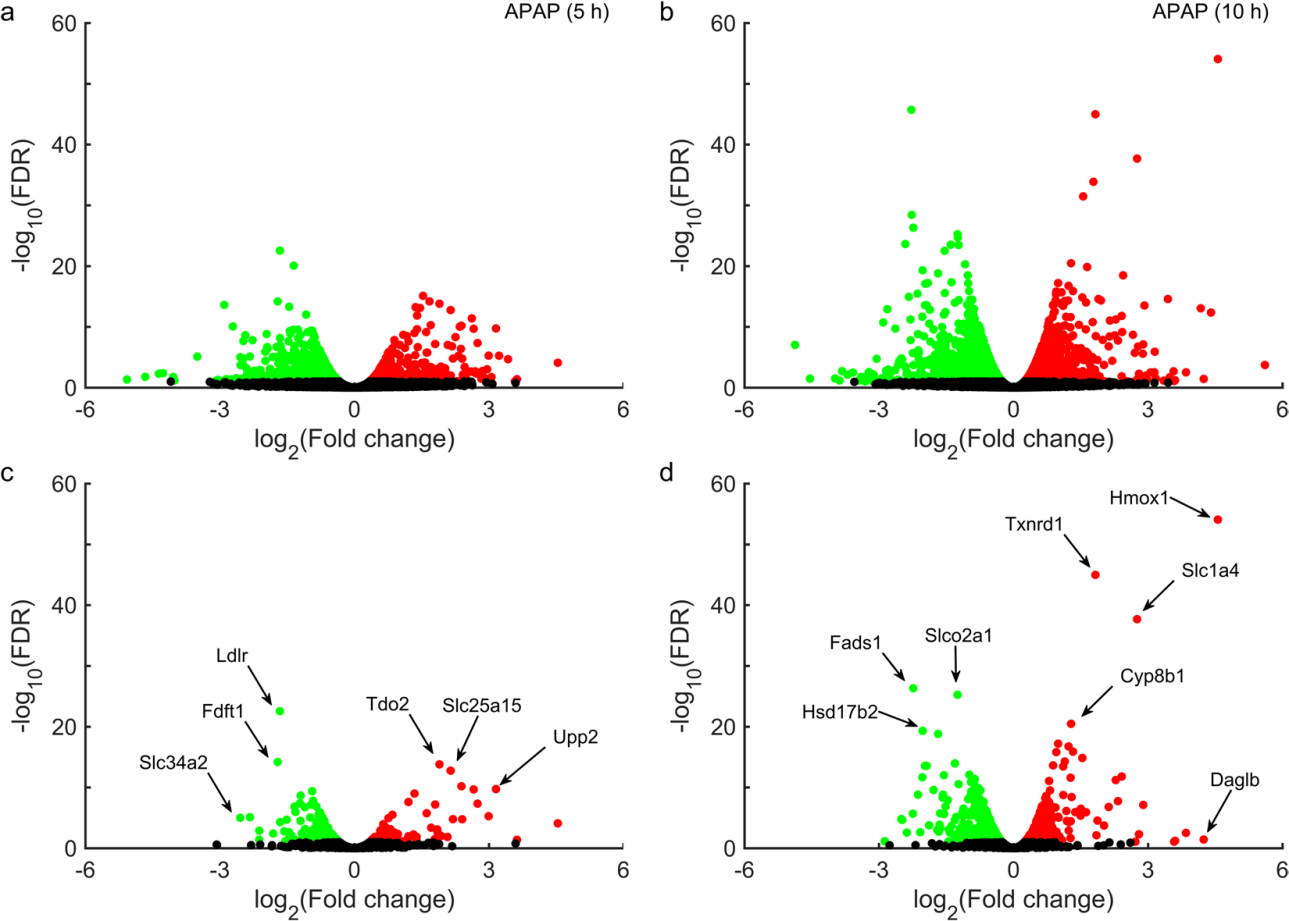
Volcano plots of differentially expressed genes (DEGs) in the liver, induced by acetaminophen (APAP). False discovery rates (FDRs) plotted against APAP-induced log_2_ fold changes in DEGs for one group of rats collected at 5 h (**a**) and a second group at 10 h (**b**). Genes from the RNA-seq data mapped onto the *iRno* model at 5 h (**c**) and 10 h (**d**). Circles in red/green show genes/transcripts that were significantly up-/down-regulated (FDR < 0.10), whereas black circles show those that were unchanged. ***Cyp8b1***: Cytochrome P450 family 8 subfamily B member 1; ***Daglb***: Diacylglycerol lipase, beta; ***Fads1***: Fatty acid desaturase 1; ***Fdft1***: Farnesyl diphosphate farnesyl transferase 1; ***Hmox1***: Heme oxygenase 1; ***Hsd17b2***: Hydroxysteroid (17-beta) dehydrogenase 2; ***Ldlr***: Low density lipoprotein receptor; ***Slco2a1***: Solute carrier organic anion transporter family, member 2a1; ***Slc1a4***: Solute carrier family 1 member 4; ***Slc25a15***: Solute carrier family 25 member 15; ***Slc34a2***: Solute carrier family 34 member 2; ***Tdo2***: Tryptophan 2,3-dioxygenase; ***Tnxrd1***: Thioredoxin reductase1; ***Upp2***: Uridine phosphorylase 2.

We used an RNA-seq analysis platform (Kallisto/Sleuth) to analyze the raw RNA sequencing reads, and identified differentially expressed genes (DEGs) by comparing transcript abundance levels between control animals and two groups of rats treated with a single dose of APAP (2 g/kg) and observed for 5 (Fig. 2a) or 10 h (Fig. 2b) after treatment. Significantly altered genes were shown using a false discovery rate (FDR) cut-off value of 0.10. The total number of significantly altered DEGs induced by APAP was lower in the 5-h group (1,384) than in the 10-h group (2,551) (Table 1). Furthermore, the observed negative logarithmic base 10 FDR values for many significant DEGs were greater than 15 for the 10-h group, indicating that APAP-induced perturbations of liver metabolism were more robust for the 10-h group than for the 5 h group.

**Table 1:** Number of differentially expressed genes (DEGs) 5 or 10 h after exposure to APAP (2 g/kg) and number of genes mapped to the *iRno* model.

Time (h)	Total number of genes	DEGs FDR < 0.10	Genes mapped to *iRno*	Mapped DEGs FDR < 0.10
5	11,721	1,384	1,710	241
10	11,659	2,551	1,701	512

FDR: false discovery rate.

Of all the unique genes identified by RNA-seq analysis, only 10% and 20% were significantly differentially expressed (FDR < 0.10) for the 5-h and 10-h groups, respectively. We used Entrez gene ID annotations and mapped all DEGs from the RNA-seq analysis onto the *iRno* model using the gene-protein-reaction annotations and identified the metabolic genes in the RNA-seq data (Fig. 2c,d). The proportion of metabolic genes from the RNA-seq analysis that mapped onto the *iRno* model (containing 2,324 total genes) was approximately 15% for both time points (Supplementary Table S1). Of these mapped genes, approximately 15% and 30% were differentially expressed significantly (FDR < 0.10) 5 (Fig. 2c) and 10 h (Fig. 2d) after APAP treatment, respectively. Our analysis suggests that, APAP induced perturbations in key metabolic genes, such as, low-density lipoprotein receptor (*Ldlr*), tryptophan 2, 3-dioxygenase (*Tdo2*), and members of the solute carrier family (*Slc25a*1*5* and *Slc34a*2) as early as 5 h post-treatment (Fig. 2c). Similarly, at 10 h post-treatment, there were major perturbations in metabolic genes related to redox metabolism, such as, heme oxygenase 1 (*Hmox*1) and thioredoxin reductase 1 (*Txnrd1*), members of the solute carrier family (*Slc1a4* and *Slco*2*a*1), and genes in the cytochrome p450 subfamily (*Cyp8b1*) (Fig. 2d). A summary of the total number of DEGs associated with APAP exposure and their corresponding mapping onto the *iRno* model is presented in Table 1. We also provide detailed results of the RNA-seq analysis for each DEG (transcript), including the p-value, FDR, logarithmic fold changes, and annotations corresponding to the genes mapped onto the *iRno* model (Supplementary Table S1).

To identify APAP-induced DEGs that lead to metabolic pathway alterations in the liver, we performed KEGG pathway enrichment analysis using the DAVID functional annotation tool^27^. We selected highly significant DEGs from among the metabolic genes mapped onto the *iRno* model based on a strict FDR cut-off of less than 0.05. Using this criterion, 5 h after APAP treatment, there was enrichment of mostly global pathways, such as, metabolic pathways and the glycerophospholipid pathway in lipid metabolism, based on an enrichment cut-off of FDR < 0.10. In contrast, 10 h post-treatment, there was significant enrichment of several other pathways, including, among others, pathways in nucleotide metabolism (pyrimidine and purine), lipid metabolism (steroid and steroid hormone biosynthesis; fatty acid degradation; and glycerolipid and glycerophospholipid metabolism), amino acid–related metabolism (tryptophan; cysteine, and methionine; glycine, serine, and threonine; arginine and proline; and glutathione metabolism) and carbohydrate metabolism (pyruvate; glyoxylate, and dicarboxylate; glycolysis/gluconeogenesis and citrate cycle). These results indicate that the APAP-induced perturbations in pathways were moderate at 5 h post-treatment. However, the majority of pathway perturbations observed at 10 h post-treatment indicated progression towards liver injury. We provide a complete summary of the KEGG-enriched pathways, along with the number of genes (and their Entrez gene IDs) mapping to each pathway and the associated significance tests in Supplementary Table S2.

### APAP treatment increases metabolic flux through pyruvate cycling and decreases glycogenolysis

**Figure 3:**
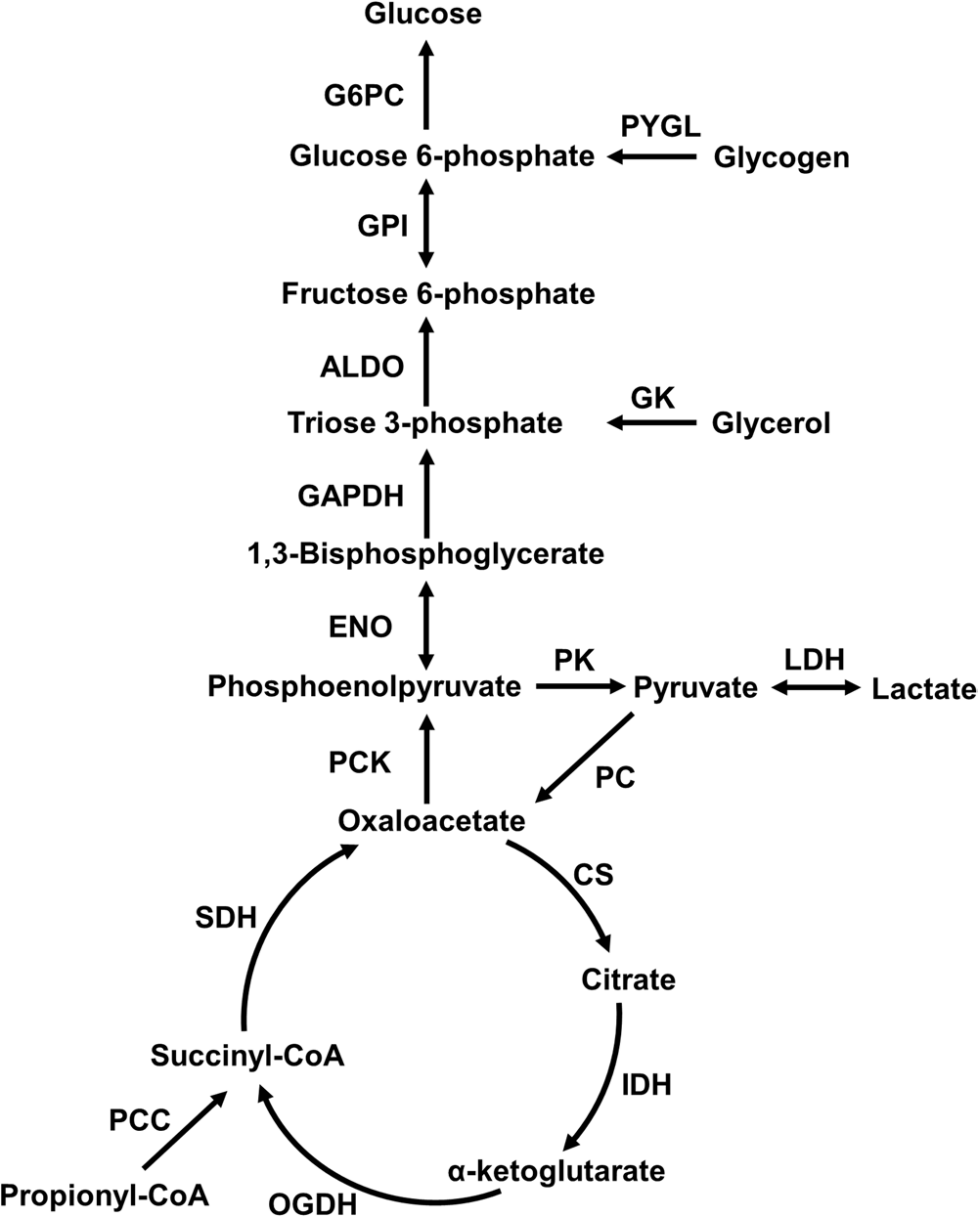
The minimal network used for estimating flux measurements in the tracer dilution study. Abbreviations shown are names of enzymes for which absolute flux was calculated based on metabolic flux analysis. **ALDO**: Aldolase; **CS**: Citrate synthase; **ENO**: Enolase; **GADPH**: Glyceraldehyde-3-phosphate dehydrogenase; **GK**: Glycerol kinase; **GPI**: D-glucose-6-phosphate isomerase; **G6PC**: D-Glucose-6-phosphatase; **IDH**: Isocitrate dehydrogenase; **LDH**: Lactate dehydrogenase; **OGDH**: Oxoglutarate dehydrogenase; **PC**: Pyruvate:carbon-dioxide ligase (ADP-forming); **PCC**: Propionyl-CoA carboxylase; **PCK**: Phosphoenolpyruvate carboxykinase; **PK**: GTP:pyruvate 2-O-phosphotransferase; **PYGL**: glycogen phosphorylase; **SDH**: Succinate dehydrogenase.

**Figure 4:**
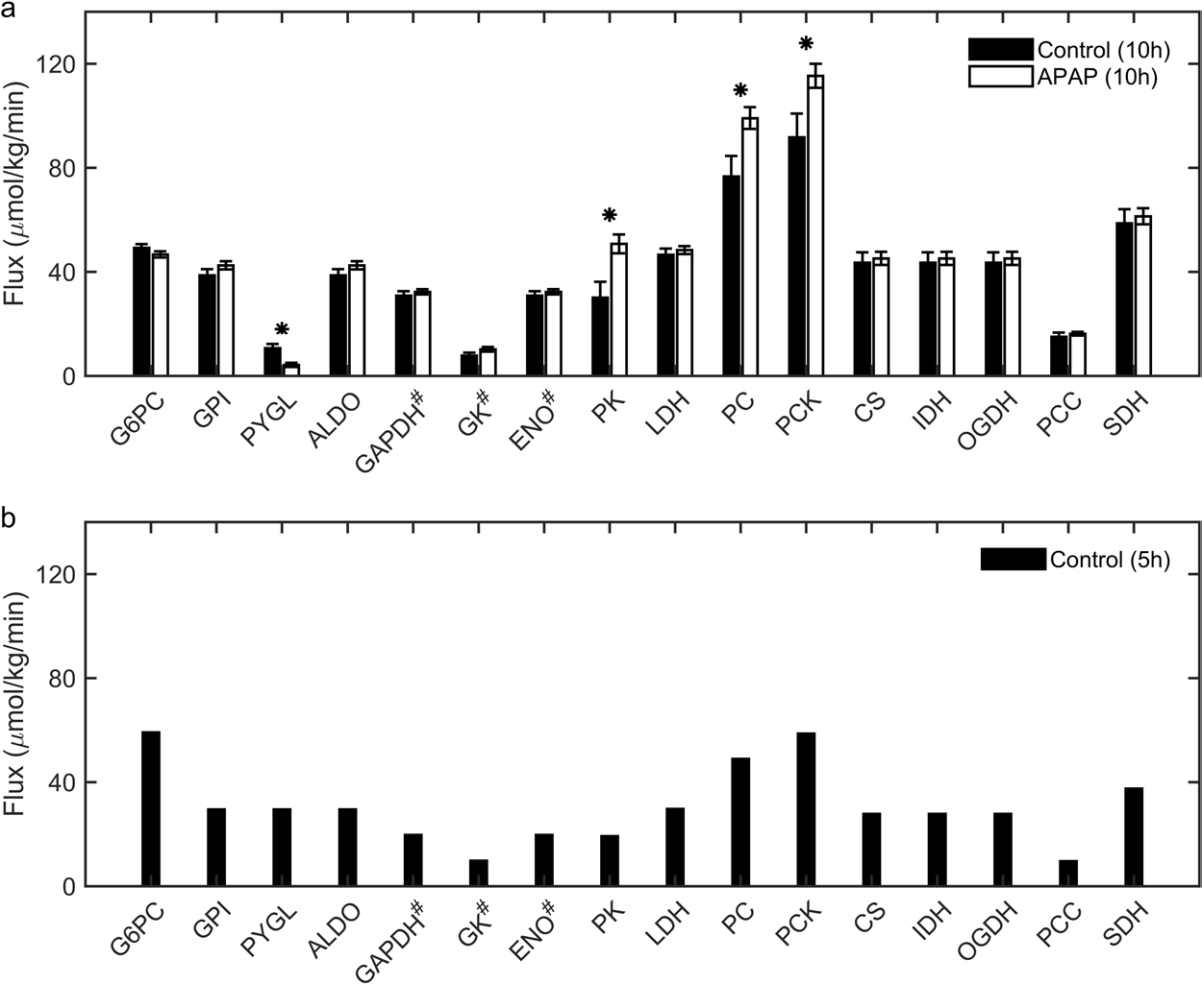
Acetaminophen (APAP)-induced absolute flux measurements in the glucose production pathway obtained from the tracer dilution study under fasting conditions. (**a**) Bar graphs of flux measurements calculated from metabolic flux analysis 10 h after treatment with APAP (unfilled bars) or vehicle (filled bars). (**b**) Bar graph of approximate absolute flux values derived from the literature for control rats studied after 5 h of fasting^31–33^ (*p < 0.05, ^#^values in Hexose units and abbreviations as in Fig. 3).

Under fasted conditions, the rat liver maintains plasma glucose levels using various gluconeogenic substrates (e.g., amino acids, lactate, and glycerol). However, APAP-induced alterations in the reaction fluxes of the glucose production pathways under such conditions are unknown. To identify these changes and thereby constrain the *iRno* model, we performed ^2^H/^13^C- tracer labeling to assess the major metabolic fluxes in the liver glucose production pathway 10 h after administration of a single dose of APAP (2 mg/kg) under fasting conditions. Using a minimal network (Fig. 3) to calculate the metabolic fluxes for the respective enzymes based on the tracer enrichment data [i.e., by applying metabolic flux analysis (MFA)], we found significant elevations in the rate of pyruvate cycling in the APAP-treated animals in comparison to control animals (Fig. 4a). The APAP-treated group also showed a significant decrease in the rate of glycogenolysis, the process of breaking down stored glycogen to produce blood glucose. However, the metabolic fluxes of other enzymes in the pathway were not significantly altered between the two groups. We previously observed these same flux changes of increased pyruvate cycling and decreased glycogenolysis when comparing two groups of mice, one subjected to long-term fasting and the other to short-term fasting^28^. Therefore, the metabolic response to APAP seems to accelerate the transition to a long-term fasting phenotype. This conclusion is supported by prior studies in mice^29^ and perfused rat livers^30^, which reported an elevated rate of glycogen depletion in response to APAP treatment.

In our experimental design, we observed rats under fasting conditions for 5 and 10 h after APAP treatment, but measured fluxes only for 10 h for the control and treatment conditions. Therefore, we calculated the approximate major fluxes in the glucose production pathway in animals fasted for 5 h (Fig. 4b) based on a survey of the literature^31–33^. Briefly, the fractional contributions of glycerol, lactate and amino acids, and glycogenolysis to liver glucose production reported in the literature were used to compute the central carbon fluxes corresponding to a twenty percent higher glucose output flux at 5 h when compared to that at 10 h^34^. We then compared the fluxes calculated at 5 h (Fig. 4b) with the measured metabolic fluxes of control animals fasted for 10 h (Fig. 4a, filled bars). As expected, glycogenolysis was the major contributor to glucose production under short-term fasting (5 h) conditions, whereas it was markedly reduced under extended fasting (10 h) conditions. During extended fasting, contributions from gluconeogenesis significantly increased (Fig. 4a). These metabolic flux measurements provide the means to integrate the gene expression changes into the *iRno* model and validate the resulting flux distributions under control conditions. Furthermore, they can also be used as further constraints in solving for metabolite alterations in the *iRno* model.

### Global metabolic changes induced by APAP and metabolite mapping to the *iRno* model

**Figure 5:**
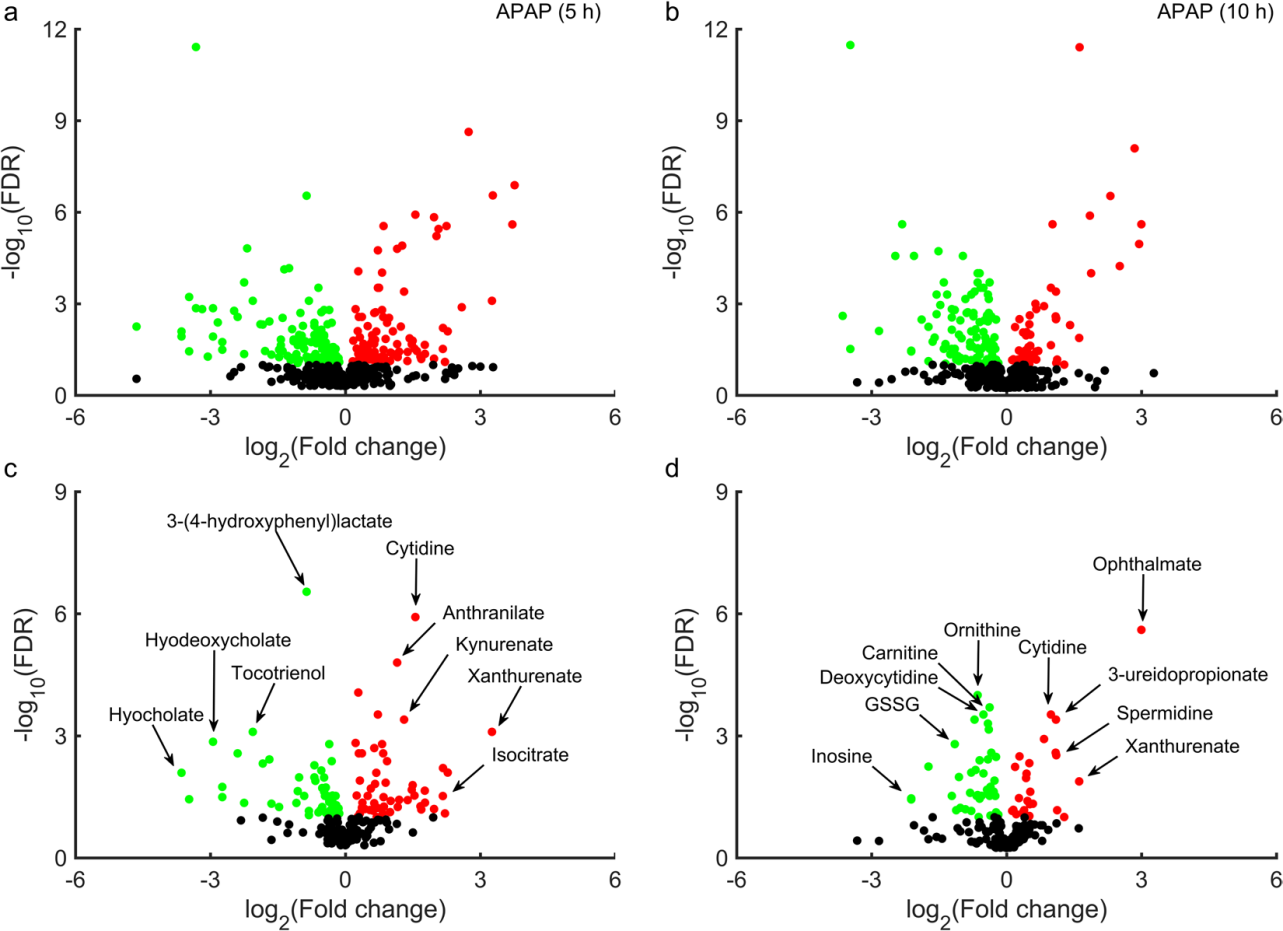
Volcano plots of global plasma metabolite changes induced by acetaminophen (APAP). False discovery rates (FDRs) plotted against APAP-induced log_2_ fold changes in plasma metabolites for one group of rats collected at 5 h (**a**) and a second group at 10 h (**b**). Metabolites mapped onto the *iRno* model based on KEGG ID annotation at 5 h (**c**) and 10 h (**d**). Red/green circles show metabolites significantly elevated/depressed (FDR < 0.10) for 5 and 10 h post APAP treatment, respectively; symbols in black show unchanged metabolites.

In order to identify the APAP-induced early perturbations in plasma metabolites that could serve as potential markers for liver injury, we collected blood samples from rats given a single dose of APAP and observed for 5 h and 10 h, and measured global metabolic profiles (Fig. 1c). We detected 569 metabolites in the plasma overall at both time points (Fig. 5a,b). Based on a relaxed FDR cut-off of less than 0.10, approximately 40% and 30% of these metabolites were significantly altered relative to controls at 5 and 10 h post-treatment, respectively. These results indicate that APAP-induced alterations in the number of plasma metabolites were higher at 5 h post-treatment and decreased at 10 h post-treatment. A two-way analysis of variance (ANOVA) of all plasma metabolites with time and treatment as the between-subject factors revealed a significant APAP-treatment effect for several metabolites (235) mapping to various metabolic pathways (Supplementary Table S3). For example, many metabolites involved in amino acid metabolism, such as those in the N-acetyl branched-chain amino acid pathway (leucine, N-acetylleucine, valine, and N-acetylvaline), significantly increased at both time points. Similarly, multiple metabolites involved in glutathione metabolism (oxidized glutathione, L-cystathionine, and cysteine) decreased due to APAP treatment.

Of all the metabolites detected in the plasma, we excluded those related to drug metabolism from analysis because the current *iRno* model does not account for them, and mapped the remaining metabolites onto the model. Because the exchange reactions for some of these mapped metabolites were not included in the original model, we further updated the model to simulate their alterations in the plasma (see Materials and Methods). Of the 569 metabolites detected, we were able to map 226 to the model based on KEGG ID annotation and name matching. Using the FDR criterion of <0.10, 103 and 67 of these 226 metabolites were significantly altered at 5 (Fig. 5c) and 10 h (Fig. 5d) after APAP treatment, respectively. Our analysis suggests that, of the total metabolites mapped onto the *iRno* model, metabolites in tryptophan metabolism (anthranilate, kynurenate, and xanthurenate) and secondary bile acid metabolism (hyocholate and hyodeoxycholate) were ranked high among the other significantly altered metabolites for the 5 h group (Fig. 5c). Similarly, metabolites in pyrimidine metabolism (cytidine, deoxycytidine, and 3-ureidopropionate) and glutathione metabolism (GSSG and ophthalmate) were ranked high among the other significantly altered metabolites for the 10-h group (Fig. 5d). We consistently observed that cytidine and xanthurenate were significantly altered at both time points among the most significantly changed metabolites. We present a complete list of mapped metabolites along with their significance levels in the Supplementary Table S4.

### Identifying transcriptomics-driven metabolite changes using the *iRno* model

**Figure 6:**
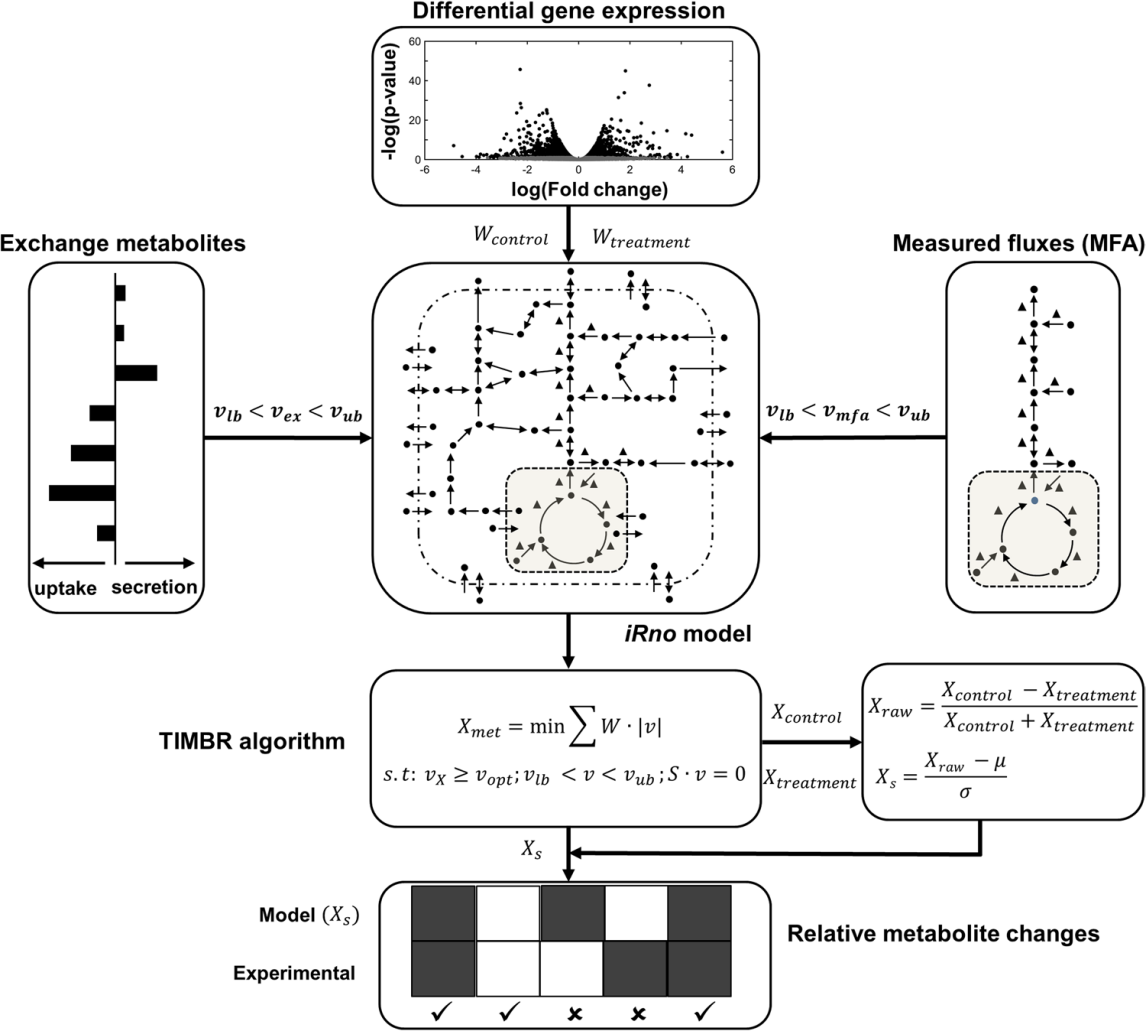
Schematic representation of how multi-omics data were integrated into the *iRno* model. The TIMBR algorithm estimates the network feasibility of producing a metabolite given changes in gene expression to the *iRno* model^16^. Here we integrated TIMBR with *in vivo* multi-omics data. We used *in vivo* differential gene expression data to determine the reaction weights (*W*) in the *iRno* model, and then used flux measurements (MFA) from a tracer labeling study (*v*_*mfa*_) as well as the physiological boundary conditions of exchange metabolites (*v*_*ex*_) to constrain the model. TIMBR then calculates the global network demand required for production of a metabolite (*X*_*met*_) by minimizing the weighted sum of flux across all reactions, under a control (*X*_*control*_) or treatment (*X*_*treatment*_) condition. We next z-transformed each raw metabolite production score (*X*_*raw*_) to calculate the TIMBR production score (*X*_*s*_) for that metabolite, which we compared with the global metabolic profiling data to assess whether its level had increased or decreased under the treatment condition relative to the control condition.

To predict metabolite alterations and elucidate the mechanism underlying their changes between control and APAP-treated conditions, we used constraint-based modeling techniques with the *iRno* model. Specifically, we integrated the APAP-induced gene expression changes in liver metabolism into the TIMBR algorithm with physiological boundary conditions, with or without the measured fluxes in the central carbon metabolism pathways (Fig. 6). Because the APAP-treatment experiments were performed under fasting conditions, we used uptake rates of amino acids, lactate, and free fatty acids from rat fasting studies in the literature (see Materials and Methods, and Supplementary Table S5). The remaining boundary conditions pertaining to essential vitamins, oxygen, and carbon dioxide were set as unconstrained as in the original *iRno* model. Furthermore, we assumed these boundary conditions to be the same for both control and APAP-treated groups.

We simulated the flux distributions of liver glucose production that would correspond to the fasting conditions, using the inputs to and outputs from the liver to constrain the *iRno* model. We predicted the central carbon metabolic fluxes that were measured via MFA, using isotope enrichment data (Figs 3 and 4a). Although the *iRno* model provided multiple solutions to generate glucose from the given inputs, we were able to obtain the measured flux distributions in the glucose production pathway in the *iRno* model by constraining their lower and upper bounds within the experimental standard deviations of the MFA data. Thus, the model simulated production of glucose, urea, and ketone bodies under fasted conditions for the given input and output conditions.

The *iRno* model cannot predict a list of plasma metabolite alterations in the absence of gene expression data. Therefore, to test its accuracy against the chance of randomly selecting this list, we randomly generated a set of normally distributed gene expression changes, which we converted to reaction weights using the TIMBR algorithm, and predicted metabolite alterations. The model predicted ~34% of the significantly altered metabolites (FDR < 0.10) observed in the plasma metabolite data (Supplementary Tables S4 and S6). This suggests that a significant portion of observed metabolite changes was embedded in the network structure itself, coupled with the physiological boundary conditions of fasting. Based on these conditions, we then evaluated the *iRno* model by providing the actual gene expression changes between control and APAP-treated groups as the inputs to identify a list of metabolite alterations for two conditions: *1*) one in which flux measurements obtained from MFA were not used as constraints (No MFA) and, *2*) another in which they were (MFA).

### MFA constraints improve metabolite predictions of the *iRno* model

**Figure 7:**
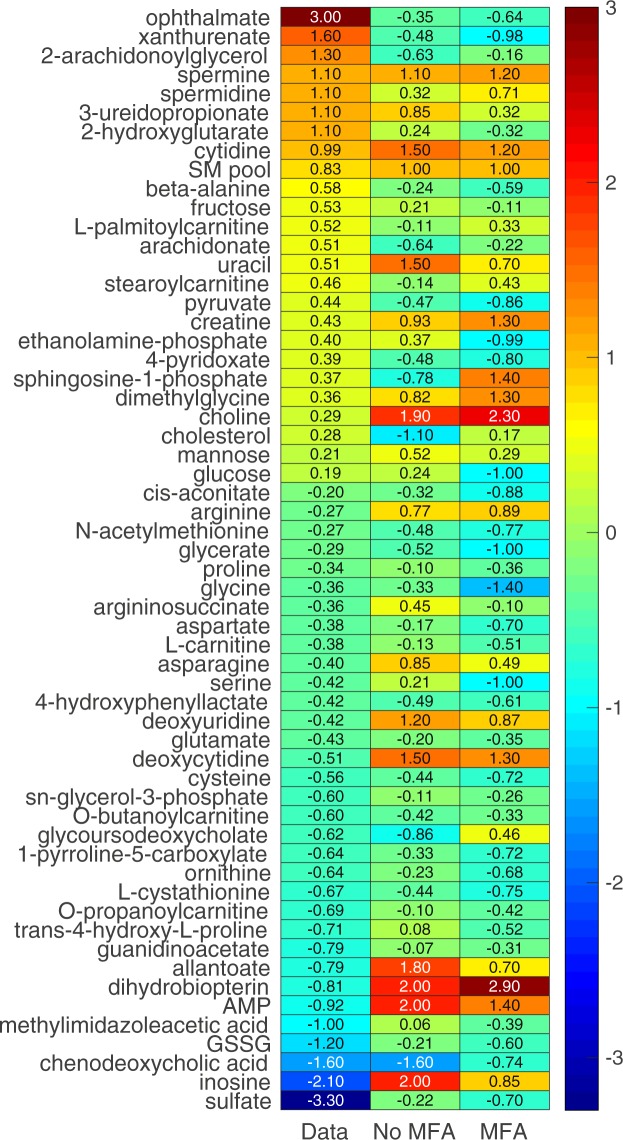
Heat map of TIMBR production scores compared to metabolic profiling data obtained 10 h after APAP treatment under fasting conditions. *iRno* model predictions calculated under two integration conditions were compared against log_2_ fold changes of metabolites that significantly (FDR < 0.10) changed in the global metabolic profiling data (Data). In one condition, only gene expression changes were used (No MFA), whereas in the other both gene expression changes and MFA data were used as constraints (MFA). The numbers in the heat map show the log_2_ fold changes of the metabolic profiling data (left column) and TIMBR production scores under the no MFA (center column) and MFA (right column) conditions. The color scheme on the far right shows the degree of change in the level of a plasma metabolite, from highly increased (dark red) to highly decreased (dark blue).

The gene expression changes obtained from RNA-seq analysis at 5 and 10 h after APAP treatment were converted to reaction weights for each control and treatment group and integrated into the TIMBR algorithm. Here, TIMBR converts all gene expression fold changes into reaction weights and inherently accounts for the significant changes, thereby avoiding the use of cut-off values that can result in loss of information (see Materials and Methods for details). Using the input and output boundary constraints under fasting conditions, we predicted TIMBR production scores, which represent altered plasma levels of metabolites that were either secreted or consumed as a result of APAP treatment. We then compared the log_2_ fold changes of metabolites identified from the global metabolic profiling analysis (Fig. 5c,d and Supplementary Table S4) with the *iRno* model predictions and assessed the accuracy of the model in predicting the direction of change in metabolite level due to APAP treatment (Fig. 7 and Supplementary Fig. S1). Of the 226 metabolites mapped onto the *iRno* model from the plasma metabolomics data, the model provided predictions for 182 at 10 h post-treatment (Supplementary Table S7). Of these, only 58 significantly changed between control and treatment conditions (FDR < 0.10). Our model correctly predicted the direction of change for 57% of the metabolites when we used gene expression changes alone to drive the TIMBR predictions with the rate of glucose secretion as the only constraint in the glucose production pathway (Fig. 7, no MFA). Similarly, when we used only the gene expression changes at 5 h post-treatment, the *iRno* model correctly predicted only 41% (Supplementary Fig. S1, No MFA) of the 95 metabolites that were experimentally changed. This reduction in model correspondence for the 5-h group may have occurred because the number of significant DEGs was lower at 5 h than at 10 h after APAP treatment (Table 1).

Interestingly, with the same input and output constraints under the fasting state, when we used the flux measurements from MFA as constraints along with the gene expression changes, our model correctly predicted the direction of change for 65% of the significantly altered metabolites (FDR < 0.10) 10 h after APAP treatment (Fig. 7, MFA). The results were slightly better (68%) when we used a less stringent criterion for comparison of significantly altered metabolites (Supplementary Fig. S2, MFA). The trend was similar when we used approximate flux values obtained from the literature for the fasting state at 5 h post-treatment: the model correctly predicted the direction of change for 53% of the altered metabolites, a 12% increase from the no MFA condition (Supplementary Fig. S1, MFA). Overall, the model predicted reductions more accurately than increases when we used only gene expression changes. When we also used MFA data, the model predicted increases more accurately than reductions for data obtained 5 h after APAP treatment. However, 10 h after treatment, when the number of significant DEGs was higher (Table 1), the model correctly predicted more than 55% of the significantly altered metabolites regardless of the direction of change. These results show that the predictions of the *iRno* model showed better correspondence with the measured metabolite changes (65%) when gene expression data at 10 h after APAP treatment were incorporated together with some of the measured fluxes as constraints. However, when the model was provided with gene expression data alone (without MFA constraints), its prediction capability decreased (57%), indicating the importance of integrating multiple data sources to obtain better predictions.

To further differentiate the metabolites that could be changing in the plasma because of changes in gene expression, we combined the highly enriched pathways obtained from KEGG pathway enrichment analysis (Supplementary Table S2) with the observed pathway-specific metabolite alterations in the data (Supplementary Table S3). Based on the KEGG pathway annotations, we identified the metabolites significantly altered at 10 h after treatment and associated them with the highly enriched pathways from the gene expression analysis (Supplementary Table S7, List of potential metabolites). Subsequent comparison of the model-predicted metabolite alterations with the metabolite alterations at the pathway level are shown in the Supplementary Table S7 (pathway-level concordance). The network model comparisons provided better predictions for the case of amino acid−related pathways compared to other pathways (such as nucleotide metabolism), as indicated by the high correlations for the gene expression-induced metabolite changes in these pathways. Overall, our data suggest that the developed framework can serve as a potential tool to integrate multiple omic datasets to identify a putative list of plasma metabolites that were associated with the toxicant-induced gene expression changes. In such an application, the predicted metabolite alterations would serve as testable hypotheses, which could be confirmed or rejected by targeted metabolomic analysis to assess the changes in individual metabolites.

## Discussion

The diverse mechanisms underlying toxicant-induced injuries of excretory organs complicate the process of identifying markers common to the detection and progression of injuries. Nevertheless, the processes involved in the initiation of such injuries and subsequent adaptations are likely to influence the metabolism of many associated endogenous substances. If so, then measuring these perturbations might help identify early toxicant-specific markers of injury progression without extensive knowledge on the toxicant’s mechanism of action. Indeed, a panel of genes was recently identified as a marker of toxic liver injuries, using gene expression patterns associated with liver histopathology in rats exposed to environmental toxicants^13^, and serum metabolites were identified as markers of acute liver toxicity^10^. To the best of our knowledge, however, no study has attempted to integrate the highly interconnected toxicant-induced perturbations of gene expression and changes in metabolic processes into a mechanistic framework to determine the extent to which gene expression changes correlate with metabolite alterations. Here, we developed an integrated experimental/computational approach using GENREs and demonstrated how changes in liver gene expression induced by APAP treatment could drive plasma metabolite alterations, which in turn can serve as the markers for liver injury.

Using results from studies on rats exposed to APAP (Fig. 1), we devised an experimental strategy that allowed us to simultaneously collect transcriptomic and metabolomic data from the same group of rats to control for inter-animal variability. A single dose of APAP treatment (2 g/kg) increased levels of ALT and AST at time points consistent with the histopathology associated with liver injury (Fig. 1a,b). However, 5 h or 10 h after APAP treatment, before ALT and AST levels were elevated; a comparison of the gene expression changes observed in the two groups showed that the number of significant DEGs increased with the time elapsed after exposure (Fig. 2). The high number of DEGs identified in the 10-h group may be due to APAP-derived intermediates causing more gene perturbations than in the 5-h group. These observations, which clearly reveal APAP-induced liver perturbations without elevated levels of ALT and AST, suggest that we can monitor these alterations to identify liver pathology.

Interestingly, heme-oxygenase 1 (*Hmox1*) was the most significantly altered gene (>23 fold change) 10 h after APAP treatment (Fig. 2d and Supplementary Table S1). In a recent study, proteomic analysis of APAP-induced hepatotoxicity identified the protein Hmox 1, the gene product of *Hmox1*, as a potential marker of liver injury^35^. However, Hmox1 was not significantly changed 5 h after APAP treatment, suggesting that the upregulation observed 10 h after treatment could reflect injury progression. Similarly, thioredoxin reductase 1 (*Txnrd1*), another key gene upregulated 10 h after APAP treatment, plays an active role in redox reactions involving hydrogen peroxide detoxification^36^ along with glutathione, which is perturbed because of acetaminophen toxicity^37^. Observations of the top three genes at both time points (Fig. 2c,d) indicate that genes in members of the solute carrier family were consistently perturbed, but more perturbations specific to acetaminophen toxicity were apparent at 10 h. A similar result was obtained with the KEGG pathway-level gene enrichment analysis, where only alterations of lipid-related metabolic pathways were observed 5 h after treatment. These modifications may be due to early changes caused by APAP-metabolism and the fasting state. At 10 h post-treatment, however, we observed changes in more focused pathways (Supplementary Table S2), including those of retinol metabolism; tryptophan metabolism; glycine, serine, and threonine metabolism; as well as cysteine and methionine metabolism. These expanded alterations 10 h after APAP treatment (Supplementary Table S2) suggest that a progressive change in molecular pathways occurred over time, with numerous pathway perturbations consistent with the known mechanisms of APAP-induced toxicity^38–40^. Specifically, high-dose APAP exposure overwhelms glutathione levels in cells, causing its depletion within 10 h after treatment in rats. Replenishment of glutathione requires increased synthesis rates for its constitutive components (i.e., cysteine, glutamate, and glycine). Consistent with this view, our analysis suggests that APAP exposure leads to early perturbations of specific metabolic pathways in the liver (e.g., glycine, serine, and threonine metabolism; cysteine and methionine metabolism), which compensate for glutathione depletion by upregulating the majority of genes involved in these pathways.

Although the APAP-induced alterations in gene expression described above are indicative of adverse effects in the liver, they cannot serve as injury markers detectable in easily accessible biofluids. In contrast, the outcomes of these gene perturbations, such as alterations in protein and metabolite levels, can serve as liver injury markers that are readily detected in plasma by existing techniques.

Our approach takes advantage of the GPR rules in GENREs and utilizes gene expression changes to predict a list of plasma metabolite alterations that can serve as potential markers for further investigation. In general, GENREs contain gaps in their networks and are iteratively and progressively updated with the latest information from the literature. In the current study, we updated the original *iRno* model and incorporated several reactions based on literature evidence (Supplementary Table S9), which increased the number of metabolites corresponding to the metabolic profiling data. We were able to make model predictions for 40% of metabolites found in the metabolic profiling data (Fig. 5c,d). Interestingly, a comparison of the directionality of changes in metabolites driven by alterations in gene expression with the measured log_2_ fold changes in metabolites indicated that the model correctly predicted the direction of change (an increase or decrease) for 65% of the metabolites 10 h after APAP treatment (Fig. 7, MFA) but only 53% 5 h after treatment (Supplementary Fig. S1, MFA), owing to the fewer APAP-induced gene perturbations observed at this time point (Table 1). When metabolic flux constraints were not used, model performance was approximately 10% lower at both time points (Fig. 7 and Supplementary Fig. S1, No MFA). These predictions highlight the existence of multiple solutions for genome-scale models and the need to incorporate more physiological constraints to yield results that are applicable and robust under *in vivo* conditions.

To ascertain the robustness of the *iRno* predictions and the contribution of each independent change in gene expression and physiological boundary condition, we performed local and global sensitivity analyses for each input category. Adding a small amount of Gaussian noise to the APAP-induced gene expression data did not alter model performance. However, as expected, adding increased random Gaussian noise reduced model performance (<45% correspondence) compared to that for unaltered gene expression data (Supplementary Table S6). Furthermore, the *iRno* model showed only ~34% correspondence when a randomly generated gene expression fold changes (from normal distribution) were used, suggesting that APAP-induced gene expression changes were indeed the driving force for the observed metabolite changes. Similarly, local and global perturbations in the boundary constraints of uptake rates reduced model correspondence with the data, showing the importance of physiological boundary conditions in improving predictions. We also investigated methods other than the TIMBR algorithm, such as a constraint-based modeling (CBM) approach based on flux variability analysis^23^, and an expression-guided flux minimization (E-Fmin) approach using the flux minimization principle^41^ for integrating gene expression changes into the *iRno* model to predict metabolite alterations. However, none of these methods yielded better results (data not shown).

Many plasma metabolites, which were significantly altered owing to APAP-induced perturbations to the liver (Supplementary Table S3), may serve as markers for liver injury. In this study, we focused on metabolite alterations that were mapped onto the *iRno* model and could be driven by changes in gene expression (Supplementary Table S4). Changes in gene expression are not perfectly correlated with protein and metabolite alterations—the final outcome of gene perturbations. Consistent with this common knowledge, our experimentally obtained gene expression changes (Tables 1 and S1) were not correlated strongly with the metabolite alterations induced at different time points following APAP treatment. Whereas the numbers of significantly altered DEGs 5 h post-treatment were lower than those 10 h after treatment, the numbers of significantly altered metabolites showed the opposite trend. In contrast, the model predictions for metabolite alterations correlated well with changes in gene expression, with model performance improving for increased numbers of significant DEGs. Overall, the model provided better predictions (~65%) when constrained by MFA data and physiological boundary conditions. However, the model predicted many false positives for metabolites that did not show experimentally significant changes. These findings suggest that gene expression changes alone are insufficient to explain all metabolite perturbations, and underscore the role of additional regulatory factors, such as posttranslational modification, gene regulation, and metabolite feedback, which result in the penultimate phenotypic response observed in plasma metabolite levels. Furthermore, although the predictions were based on satisfying the physiological constraints for only liver metabolism, the observed plasma metabolite alterations could have resulted from non-transcription–driven metabolite alterations in different organs. Hence, TIMBR scores tend to overestimate actual physiological changes leading to false positives. Improvements in the experimental design, such as, measuring plasma metabolite changes directly from the liver circulation, and incorporating further details into GENREs, and considering additional information on the physiological conditions under which these modifications occur, might improve the model predictions.

Despite the aforementioned limitations, we predicted many metabolite alterations consistent with the global metabolic profiling data. To identify metabolites whose alterations were strongly correlated with gene expression changes, we further applied a selection criterion, where we used toxicant-induced pathway-level gene perturbations and identified significantly altered metabolites within them together with GENRE predictions. A comparison of model predictions against metabolites in the highly changed pathways resulted in far greater concordance with respect to specific amino-acid related pathways (Supplementary Table S7). Using this, for extended APAP treatment, the model correctly predicted highly increased levels of plasma metabolites, such as spermine, spermidine, 3-ureidopropionate, and cytidine (Fig. 7 and Supplementary Table S7). Similarly, the model correctly predicted many highly decreased metabolites, such as cysteine, glycine, serine, glutathione disulfide, and ornithine. Furthermore, our model predictions were consistent at both time points (5 and 10 h after APAP treatment) for a set of altered plasma metabolites, including cytidine, the sphingomyelin pool, uracil, choline, proline, glycine, trans-4-hydroxy-L-proline, and chenodeoxycholic acid, some of which are known plasma markers of APAP-induced liver injury^14,42–44^. Based on our analysis, we have provided a potential list of metabolites that could be highly correlated with gene expression changes (Supplementary Table S7). Our results suggest more focused experiments that can be designed in the future to identify other plasma metabolite markers of APAP-induced liver injury.

In summary, using changes in gene expression induced by acetaminophen—an exemplar liver toxicant—we identified GENRE-driven metabolite changes in the plasma and compared the model outcomes with those of global metabolic profiling data extracted under the same conditions. We found that the model predictions were much better than a random chance when using gene expression changes alone as the input. However, adding more physiological information, such as measured fluxes in the central carbon metabolism pathways, considerably improved the model predictions. The model predictions for metabolite alterations were consistent with the APAP-induced changes in liver gene expression after short (5 h) and extended (10 h) exposures, with the latter yielding more gene perturbations in greater concordance with metabolite alterations. Furthermore, we identified metabolite alterations—driven by changes in gene expression consistent with the model predictions—which can be further evaluated to assess their ability to serve as markers of APAP-induced liver injury. Our results suggest that the platform developed here, could serve as a tool in the initial step of identifying putative plasma metabolites as markers of toxicant-induced organ injuries, and has the potential to be applied broadly to other studies in drug development and metabolite marker discovery.

## Materials and Methods

### Animals

Male Sprague-Dawley rats, 10 weeks of age, were purchased from Charles River Laboratories (Wilmington, MA). The rats were fed with Formulab Diet 5001 (Purina LabDiet; Purina Miles, Richmond, IN) and were given water *ad libitum* in an environmentally controlled room, set at 23 °C and on a 12:12-h light-dark cycle. All experiments were conducted in accordance with the *Guide for the Care and Use of Laboratory Animals* of the United States Department of Agriculture and the National Institutes of Health, and all protocols were approved by the Vanderbilt University Institutional Animal Care and Use Committee, and the U.S. Army Medical Research and Materiel Command Animal Care and Use Review Office. The investigators adhered to the Animal Welfare Act Regulations and other Federal statutes relating to animals and experiments involving animals.

### Experimental Design

Surgery for implanting the catheters was performed 7 days before each experiment as previously described^45^. Rats were anesthetized with isoflurane. For studies to determine the appropriate APAP dose and exposure time and those to measure changes in gene expression and plasma metabolite profiles, the right external jugular vein was cannulated with sterile silicone catheters (0.51 mm inner diameter [ID]/0.94 mm outer diameter [OD]). For studies to measure metabolic flux, the carotid artery and the right external jugular vein were cannulated with sterile silicone catheters (0.51 mm ID/0.94 mm OD). The free end of the catheter was passed subcutaneously to the back of the neck where it was fixed. The catheter was occluded with a metal plug following a flush of heparinized saline (200 U heparin/ml). After surgery, rats were housed individually.

#### Preliminary studies for determining appropriate dose and exposure time

Two days before each study, the rats were moved from their regular housing cages to metabolic cages (Harvard Apparatus, Holliston, MA). To determine the appropriate dose and exposure time of APAP, they were treated with either vehicle (6 ml/kg of 50% polyethylene glycol, n = 6) or either 1 g/kg (n = 6) or 2 g/kg (n = 7) of APAP at 7 a.m. by gavage. Blood and accumulated urine were collected at 7 a.m. and 5 p.m. daily for 3 days.

#### Studies for measuring changes in gene expression and plasma metabolite profiles

We chose 2 g/kg as the appropriate APAP dose and two exposure times, one short (5 h, n = 8) and the other long (10 h, n = 8), based on the results of the dose-response study (Fig. 1a,b). Following blood collection, animals were given either vehicle or APAP by gavage at 7 a.m. and moved to new housing cages, where they could access water *ad libitum* but were not given food. At 12 p.m. (5-h group) or 5 p.m. (10-h group), after blood was collected from each group, animals were anesthetized by an intravenous injection of sodium pentobarbital through the jugular vein catheter and a laparotomy was performed immediately. The liver was dissected and frozen using Wollenberger tongs precooled in liquid nitrogen. The collected plasma was kept in a −80 °C freezer prior to analyses.

#### Studies for measuring metabolite flux

For flux measurements, at 7 a.m. on the day of the study, rats were administered either APAP (2 g/kg, n = 8) or vehicle (50% polyethylene glycol, 6 ml/kg, n = 8) by oral gavage, and food and water were subsequently removed. At 12:50 p.m., they were anesthetized with isoflurane, and following collection of 200 µl of arterial blood through the carotid artery catheter to determine the natural isotopic abundance of circulating glucose, a bolus of [^2^H]_2_water (99.9%) was delivered subcutaneously to enrich total body water to 4.5%. At 1 p.m. (i.e., 6 h after dosing), after they had recovered from anesthesia, the rats were placed in bedded containers without food or water and connected to sampling and infusion lines. A prime-constant infusion of [6,6–^2^H_2_]glucose (80 mg/kg prime + 0.8 mg/kg/min infusion) was administered into the systemic circulation through the jugular vein catheter for the duration of the study. Sodium [^13^C_3_]propionate (99%) was delivered as a prime-constant infusion (110 mg/kg + 5.5 mg/kg/min infusion) starting 120 min after the [^2^H]_2_water bolus. All infusates were prepared in a 4.5% [^2^H]_2_water-saline solution unless otherwise specified. Stable isotopes were obtained from Cambridge Isotope Laboratories (Tewksbury, MA). Blood glucose was monitored (AccuCheck, Roche Diagnostics, Indianapolis, IN) and donor erythrocytes were infused to maintain hematocrit throughout the study. Three blood samples (300 μl each) were collected over a 20-min period following 100 min of [^13^C_3_] propionate infusion. Arterial blood samples were centrifuged in EDTA-coated tubes for plasma isolation, and the three 100 μl plasma samples were stored at −20 °C prior to glucose derivatization and gas chromatography-mass spectrometry (GC-MS) analysis. Rats were rapidly euthanized through the carotid artery catheter immediately after the final steady-state sample.

#### Preparation of glucose derivatives

Plasma samples were divided into three aliquots and derivatized separately to obtain di-*O*-isopropylidene propionate, aldonitrile pentapropionate, and methyloxime pentapropionate derivatives of glucose. For di-*O*-isopropylidene propionate preparation, proteins were precipitated from 20 µl of plasma using 300 µl of cold acetone, and the protein-free supernatant was evaporated to dryness in screw-cap culture tubes. Derivatization proceeded as described previously^46^ to produce glucose 1,2,5,6-di-isopropylidene propionate. For aldonitrile and methyloxime derivatization, proteins were precipitated from 10 µl of plasma using 300 µl of cold acetone, and the protein-free supernatants were evaporated to dryness in microcentrifuge tubes. Derivatizations then proceeded as described previously^46^ to produce glucose aldonitrile pentapropionate and glucose methyloxime pentapropionate. All derivatives were evaporated to dryness, dissolved in 100 µl of ethyl acetate, and transferred to GC injection vials with 250-µl glass inserts for GC-MS analysis.

### Measurement of tissue injury markers in blood

Plasma levels of ALT and AST were measured using ALT and AST activity assay kits (Sigma-Aldrich, St Louis, MO), respectively.

### GC-MS analysis

GC-MS analysis was performed using an Agilent 7890 A GC system with an HP-5 ms (30 m × 0.25 mm × 0.25 μm; Agilent J&W Scientific) capillary column interfaced with an Agilent 5975 C Mass Spectrometer. Samples were injected into a 270 °C injection port in splitless mode. Helium flow was maintained at 0.88 ml∙min^−1^. For analysis of di-*O*-isopropylidene and aldonitrile derivatives, the column temperature was held at 80 °C for 1 min, ramped up at 20 °C∙min^−1^ to 280 °C and held for 4 min, then ramped up at 40 °C∙min^−1^ to 325 °C. For methyloxime derivatives, the same program was used except the ramp up to 280 °C was 10 °C∙min^−1^. After a 5 min solvent delay, the mass spectrometer collected data in scan mode from m/z 300 to 320 for di-*O*-isopropylidene derivatives, m/z 100 to 500 for aldonitrile derivatives, and m/z 144 to 260 for methyloxime derivatives. Each derivative peak was integrated using a custom MATLAB function^47^ to obtain mass isotopomer distributions (MIDs) for six specific ion ranges: aldonitrile - m/z 173–177, 259–265, 284–288, 370–374; methyloxime - m/z 145–149; di-*O*-isopropylidene - m/z 301–308. To assess uncertainty, the root mean squared error was calculated by comparing the baseline MID of unlabeled glucose samples with the theoretical MID computed from the known abundances of naturally occurring isotopes.

### ^2^H/^13^C metabolic flux analysis (MFA)

The *in vivo* MFA methodology employed in these studies has previously been described in detail^48^. Briefly, a reaction network was constructed using the INCA software package^49^ (http://mfa.vueinnovations.com/mfa). This network defined the carbon and hydrogen transitions for biochemical reactions linking hepatic glucose production and associated intermediary metabolic reactions. The flux through each reaction was estimated relative to citrate synthase (fixed at 100) by minimizing the sum of squared residuals between the simulated and experimentally determined MIDs of the six fragment ions previously described. Flux estimation was repeated 25 times from random initial values. Goodness-of-fit was assessed by the chi-square test, and 95% confidence intervals were computed by evaluating the sensitivity of the sum-of-squared residuals to variations in flux values^50^. The average SSR of each experimental group fell within the 99% confidence interval of the corresponding chi-square distribution with 22 degrees of freedom (i.e., the regressions were overdetermined by 22 measurements). Control SSR: 29.65 ± 7.05; APAP SSR: 41.87 ± 2.47. 99% CI = [8.6, 42.8]. Relative fluxes were converted to absolute values using the known [6,6-^2^H_2_]glucose infusion rate and rat weights. Flux estimates for the steady-state samples were averaged to obtain a representative set of values for each rat.

### Metabolomic analysis

Sample preparation was carried out at Metabolon Inc. (Durham, NC), in a manner similar to a previous study^51^. Briefly, individual samples were subjected to methanol extraction and then split into aliquots for analysis by ultrahigh performance liquid chromatography/MS (UHPLC/MS). The global biochemical profiling analysis comprised four unique arms, consisting of reverse-phase chromatography positive ionization methods optimized for hydrophilic compounds (LC/MS Pos Polar) and hydrophobic compounds (LC/MS Pos Lipid), reverse-phase chromatography with negative ionization conditions (LC/MS Neg), as well as a hydrophilic interaction liquid chromatography (HILIC) method coupled to negative ionization (LC/MS Polar)^52^. All methods alternated between full scan MS and data-dependent MS^*n*^ scans. The scan range varied slightly between methods but generally covered 70–1000 *m*/*z*.

Metabolites were identified by automated comparison of the ion features in the experimental samples to a reference library of chemical standard entries that included retention time, molecular weight (*m/z*), preferred adducts, and in-source fragments as well as associated MS spectra, and curated by visual inspection for quality control using software developed at Metabolon. Identification of known chemical entities was based on comparison to metabolomic library entries of purified standards^53^.

Two types of statistical analyses were performed: *1*) significance tests and *2*) classification analysis. Standard statistical analyses were performed in ArrayStudio on log‐transformed data. The R program (http://cran.r‐project.org) was used for non-standard analyses. Following log transformation and imputation of missing values, if any, with the minimum observed value for each compound, Welch’s two-sample t-test was used to identify biochemicals that differed significantly (*p* < 0.05) between experimental groups. An estimate of the FDR (*q*‐value) was calculated to take into account the multiple comparisons that normally occur in metabolomics‐based studies.

### RNA isolation and sequencing

Frozen whole liver was powdered in liquid nitrogen. Total RNA was isolated from the liver using TRIzol Reagent (Thermo Fisher Scientific, Waltham, MA) and the direct-zol RNA MiniPrep kit (Zymo Research, Irvine, CA). The isolated RNA samples were then submitted to the Vanderbilt University Medical Center VANTAGE Core (Nashville, TN) for RNA quality determination and sequencing. Total RNA quality was assessed using a 2100 Bioanalyzer (Agilent, Santa Clara, CA). At least 200 ng of DNase-treated total RNA with high RNA integrity was used to generate poly-A-enriched mRNA libraries, using KAPA Stranded mRNA sample kits with indexed adaptors (Roche, Indianapolis, IN). Library quality was assessed using the 2100 Bioanalyzer (Agilent), and libraries were quantitated using KAPA library Quantification kits (Roche). Pooled libraries were subjected to 75-bp single-end sequencing according to the manufacturer’s protocol (Illumina HiSeq3000, San Diego, CA). Bcl2fastq2 Conversion Software (Illumina) was used to generate de-multiplexed Fastq files.

### Analysis of RNA-seq data

We analyzed RNA-seq data with Kallisto, a recently developed RNA-seq data analysis tool for read alignment and quantification. Kallisto pseudo-aligns reads to a reference, producing a list of transcripts that are compatible with each read while avoiding alignment of individual bases^54^. In this study, we pseudo-aligned the reads to the rat transcriptome downloaded from the Kallisto web-site (http://bio.math.berkeley.edu/kallisto/transcriptomes). Kallisto achieves a level of accuracy similar to that of other methods but is orders of magnitude faster; this allows calculation of the uncertainty of transcript abundance estimates, via the bootstrap technique of repeating analyses after resampling with replacement from the data. Here we employed bootstrapping by repeating analyses 100 times with resampling for each data set. Considering that the average number of reads per data set is 35 million (25 to 51 million single-end reads), using other software tools to perform the same bootstrap analysis becomes prohibitively expensive.

To identify DEGs from transcript abundance data quantified by Kallisto, we used the companion tool Sleuth, which uses the results of the bootstrap analysis during transcript quantitation to estimate the technical variance directly for each sample^55^. Many software tools for differential gene expression analysis of RNA-seq experiments assume that the technical variance of gene counts follows a Poisson distribution, in which the variance equals the mean^56^. However, for many genes, the technical variance can be much higher than the expected Poisson variance^57^. A distinct advantage of Sleuth is that it models biological and technical variances explicitly with a response error model.

To understand the biological significance of the lists of genes whose expression levels were altered by APAP exposure, we used the DEGs derived from Kallisto-Sleuth analyses and identified significantly altered DEGs that were mapped to the rat GENRE, and used KEGG pathways to identify molecular pathways that were significantly enriched. We used the online tool Database for Annotation, Visualization, and Integrated Discovery (DAVID)^27^ to perform this task.

### Rat GENRE and model curation

We reconstructed a functional rat GENRE (*iRno*), using orthology annotations from genes in the Human Metabolic Reaction 2 (HMR2) database^58^, and manually reconciled several reactions by referring to the experimental literature and annotation databases^16^. The developed model, which contains 2,324 genes and 5,620 metabolites in 8,268 reactions connected by GPR rules, was validated for simulating 327 liver-specific metabolite functions successfully representing liver metabolism^16^. In this work, we further updated the *iRno* by incorporating new reactions or modifying some of the existing reactions based on experimental evidence (Supplementary Table S8). For example, although the pyruvate kinase reaction (EC: 2.7.1.40) was reported as a reversible reaction in the original model, the Gibb’s free energy of the reaction under physiological conditions suggests that it is irreversible^59^. Similarly, for the heme:oxygen oxidoreductase reaction (EC: 1.14.14.18), we corrected the substrates and stoichiometry of the reaction components for consistency^60^. Using the metabolite evidence from the global metabolic profiling of plasma samples in the current study, we added 90 transport and 105 exchange reactions to the original model to increase the number of metabolites mapping to the data. We provide the updated *iRno* model with these modifications in Supplementary Table S9.

### Boundary conditions for *iRno* in the fasting state

Our experimental design involved subjecting rats to APAP treatment under fasting conditions to maintain similar weight loss in the control and treatment groups. During fasting, the liver takes up gluconeogenic substrates, such as amino acids (AAs), lactate, and glycerol, to produce blood glucose, urea, and ketone bodies and takes up free fatty acids (FAs) for energy maintenance. Thus, the input fluxes (uptake rates) to our model are those of *1*) AAs, *2*) lactate, and *3*) FAs and glycerol. The output fluxes (secretion rates) are those of *1*) glucose derived from glycogenolysis and gluconeogenesis, as well as *2*) urea and ketone bodies.

Multiple studies have used rats fasted overnight to deplete glycogen and measured input (AAs and lactate) and output (urea and ketone bodies) fluxes, using liver perfusion and *in situ* MFA^61–66^. In these studies, sham-treated control animals subjected to fasting conditions showed significant uptake rates of AAs and lactate with subsequent production of glucose, urea, and ketone bodies^62^. Furthermore, most of these studies measured the uptake/secretion rates from rats fasted for about 24 h and evaluated the metabolic state of the liver under *ex vivo* perfusion conditions^61,62,64–66^. We noted considerable inconsistency in the uptake rates reported in these studies (Supplementary Table S5). In contrast, the study by Izamis *et al*.^63^ measured uptake/secretion rates from rats fasted overnight and used metabolite concentrations and flow rates in the major vessels entering and leaving the liver under *in situ* conditions to evaluate the metabolic state of the liver. These conditions were similar to those in our experimental design. Thus, we used the majority of the approximated uptake and secretion rates derived from the study by Izamis *et al*. to constrain the respective input and output conditions for simulating metabolite alterations. In doing so, we strictly enforced the values for all of the uptake rates by constraining the lower and upper bounds in our model, while we constrained the values for the secretion rates only in terms of the lower bounds. We provide a detailed summary of the uptake and secretions rates from these studies in Supplementary Table S5.

### Transcriptionally inferred metabolic biomarker response (TIMBR) algorithm for metabolite predictions

TIMBR is a novel method used for predicting toxicant-induced perturbations in metabolites by integrating gene expression changes into GENREs^16^. Briefly, it converts log_2_ fold changes of all DEGs into weights (*W*) for each of the GPRs in the GENRE. These reaction weights are then transformed into larger (or smaller) weights to represent relative levels of expression between the control and toxicant-treated conditions. TIMBR then calculates the global network demand required for producing a metabolite (*X*_*met*_) by minimizing the weighted sum of fluxes across all reactions for each condition and metabolite, so as to satisfy the associated mass balance and an optimal fraction of maximum network capability (*v*_*opt*_) to produce a metabolite as follows^16^ (see ref.^16^ for details):

1 $$\begin{array}{c}{X}_{met}=\,{\rm{\min }}\,\sum W\cdot |v|\\ s.t.:{v}_{X}\ge {v}_{opt};\,{v}_{lb} < v < {v}_{ub};S\cdot v=0\end{array}$$

where *W* denotes the vector representing the reaction weights, *v* is a vector of reaction fluxes, and *S* is the stoichiometric matrix. We integrated the aforementioned boundary conditions for uptake and secretion rates into the algorithm by fixing the respective lower (*v*_*lb*_) and upper bounds (*v*_*ub*_) of the exchangeable reactions (*v*_*ex*_) in the model (Eq. 2). Similarly, we integrated measurements from the ^13^C-labeled tracer studies for some of the central carbon metabolism fluxes into the TIMBR algorithm by constraining the lower and upper bounds of the respective reactions in the model (*v*_*mfa*_) (Eq. 3).

2 $${v}_{lb} < {v}_{ex} < {v}_{ub}$$

3 $${v}_{lb} < {v}_{mfa} < {v}_{ub}$$

Using this method, we determined the relative production scores for all metabolites (*X*_*raw*_) from control (*X*_*control*_) and toxicant-treated (*X*_*treatment*_) conditions (Eq. 4), and then calculated the TIMBR production scores (*X*_*s*_) as the z-transformed scores across all exchangeable metabolites (Eq. 5).

4 $${X}_{raw}=\frac{{X}_{control}-{X}_{treatment}}{{X}_{control}+{X}_{treatment}}$$

5 $${X}_{s}=\frac{{X}_{raw}-\mu }{\sigma }$$

The schematic in Fig. 6 depicts the overall integration strategy. More detailed descriptions of the TIMBR algorithm and the corresponding codes are available in the original publication^16^.

We used the experimental log_2_ fold changes of significantly altered (FDR < 0.10) plasma metabolites from the global metabolic profiling data (Supplementary Table S3) and then compared the corresponding *iRno* model predictions under no MFA and MFA conditions 5 or 10 h after APAP treatment (Supplementary Tables S4 and S7). Here, the model predictions of altered metabolite levels were considered as having increased or decreased based on TIMBR production score cut-off values of greater than 0.1 and less than −0.1, respectively. Metabolites with scores that were between −0.1 and 0.1 were considered as unchanged.

### Data and code availability

Normalized gene expression data from the RNA-seq analysis and genes mapped to the *iRno* model are provided in Supplementary Table S1. The results of KEGG pathway enrichment analysis using the mapped genes are provided in Supplementary Table S2. The results from global metabolic profiling are provided in Supplementary Table S3. Metabolites mapped to *iRno* model are provided in Supplementary Table S4. The physiological boundary constraints required to simulate the metabolite predictions are provided in Supplementary Table S5. TIMBR predictions under random gene expression changes and addition of noise to the gene expression changes are provided in Supplementary Table S6. TIMBR predictions (Figs 7 and S1) are provided in Supplementary Table S7. Details of the modifications made to the *iRno* model are provided in Supplementary Table S8. An Excel file of the updated *iRno* model is provided in Supplementary Table S9. Additional information required to reproduce the figures can be obtained via the code made available as part of this publication at https://github.com/BHSAI/APAP_toxicity_liver. Detailed explanations for TIMBR algorithm are available as part of the original TIMBR publication^16^ at www.github.com/csbl/ratcon1.

## Electronic supplementary material

Supplementary Table S1

Supplementary Table S2

Supplementary Table S3

Supplementary Table S4

Supplementary Table S5

Supplementary Table S6

Supplementary Table S7

Supplementary Table S8

Supplementary Table S9

Supplementary Information
