# Supplementary material for: Metabolic network-based predictions of toxicant-induced metabolite changes in the laboratory rat
Source: Sci Rep. 2018 Aug 3;8:11678. doi: 10.1038/s41598-018-30149-7 (PMC6076258; doi:10.1038/s41598-018-30149-7)
Supplement: Supplementary file 10 — Supplementary Information [file 41598_2018_30149_MOESM10_ESM.pdf]

Supplementary Information

Metabolic network-based predictions of toxicant-induced metabolite changes in the laboratory rat

Venkat R. Pannala[1, *], Martha L. Wall[3], Shanea K. Estes[2], Irina Trenary[3], Tracy P. O'Brien[2], Richard L. Printz[2], Kalyan C. Vinnakota[1], Jaques Reifman[1], Masakazu Shiota[2], Jamey D. Young[2, 3, *], Anders Wallqvist[1, *]

[1]Department of Defense Biotechnology High Performance Computing Software Applications Institute, Telemedicine and Advanced Technology Research Center, U.S. Army Medical Research and Materiel Command, Fort Detrick, MD 21702, USA

[2]Department of Molecular Physiology and Biophysics, Vanderbilt University School of Medicine, Nashville, TN 37232, USA

[3]Department of Chemical and Biomolecular Engineering, Vanderbilt University School of Engineering, Nashville, TN 37232, USA

[*]To whom correspondence should be addressed:

Anders Wallqvist.

Phone: 301-619-1989, Fax: 301-619-1983

E-mail: sven.a.wallqvist.civ@mail.mil

Jamey D. Young

Phone: 615-343-4253, Fax: 615-343-7951

Email: j.d.young@vanderbilt.edu

Venkat R. Pannala

Phone: 301-619-1926, Fax: 301-619-1983

E-mail: vpannala@bhsai.org

Supplementary Tables

Supplementary Table S1: Summary of gene expression changes in rats 5 or 10 after being exposed to a single dose of acetaminophen (2 g/kg).

Supplementary Table S2: A detailed summary of KEGG pathways enriched by differentially expressed genes (FDR < 0.05) in rats 5 or 10 h after being exposed to an acute dose of acetaminophen.

Supplementary Table S3: Detailed information on all plasma metabolites whose levels were altered in rats 5 or 10 h after being exposed to an acute dose of acetaminophen.

Supplementary Table S4: List of metabolites mapped onto the *iRno* model from the global metabolic profiling data.

Supplementary Table S5: Physiological boundary conditions of uptake and secretion rates for the rat liver under fasting conditions

Supplementary Table S6: TIMBR predictions for metabolite alterations based on random gene expression changes. Sensitivity of TIMBR production scores for candidate plasma metabolite markers of gene expression changes (with transcriptional noise added) induced in rats 10 h after acetaminophen treatment.

Supplementary Table S7: TIMBR production scores for candidate plasma metabolite markers of gene expression changes induced in rats 5 or 10 h after being exposed to an acute dose of acetaminophen. *iRno* model concordance based on pathway-level analysis for the 10 h time point.

Supplementary Table S8: List of updated reactions added to the stoichiometric matrix and modifications made to the reaction annotations in the *iRno* model.

Supplementary Table S9: Updated *iRno* metabolic genome-scale network reconstruction in Excel format.

Supplementary Figures

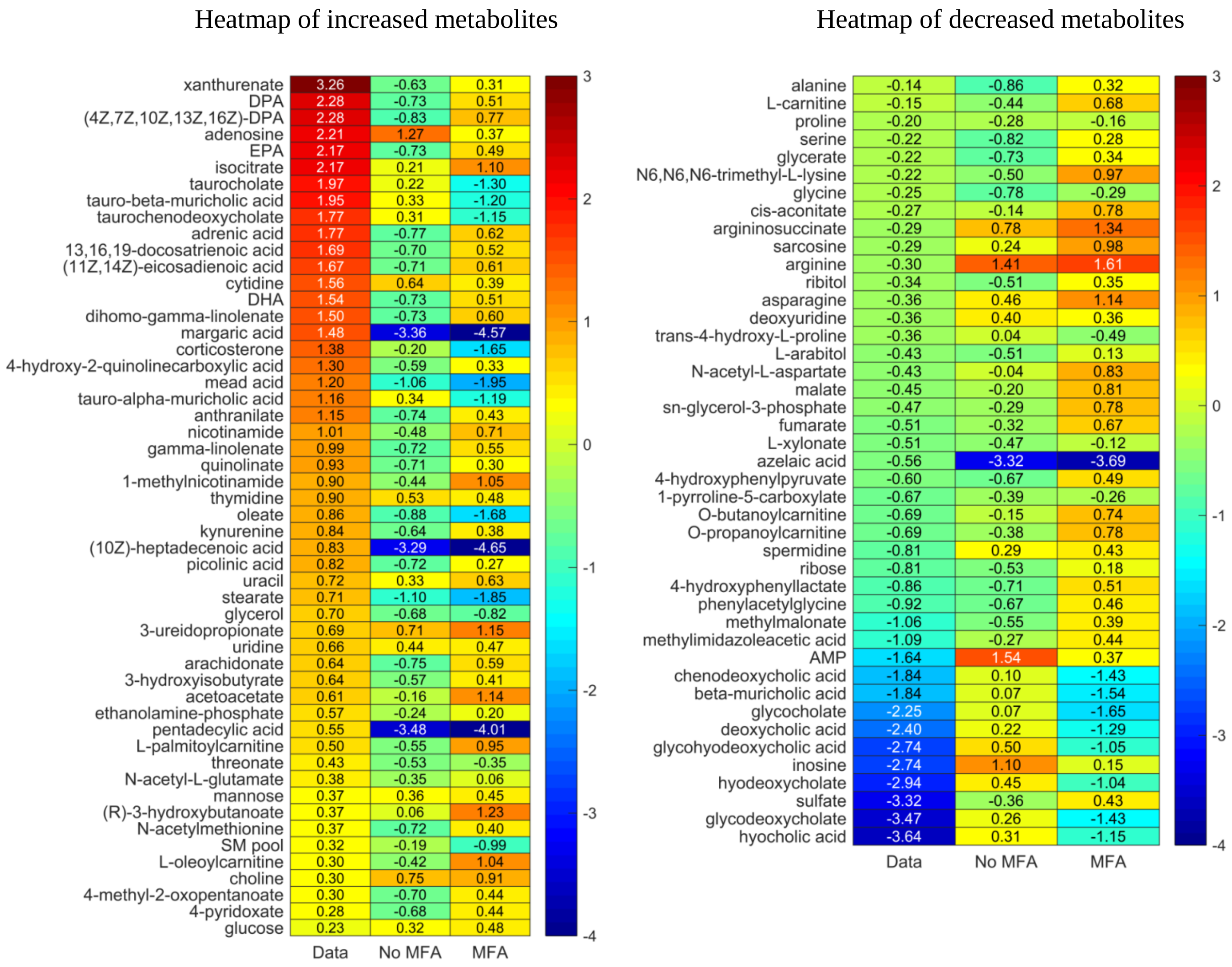

Supplementary Figure S1: Heat map of TIMBR production scores compared to metabolic profiling data obtained 5 h after APAP treatment under fasting conditions. *iRno* model predictions calculated under two conditions were compared against $\log_2$ fold changes of metabolites that significantly changed in the global metabolic profiling data (Data). In one condition, only gene expression changes were used (no MFA), whereas in the other both gene expression changes and MFA data were used as constraints (MFA). The numbers in the heat map show the $\log_2$ fold changes of the metabolic profiling data (left column) and TIMBR production scores under the no MFA (center column) and MFA (right column) conditions. The color scheme on the far right shows the degree of change in the level of a plasma metabolite, from highly increased (dark red) to highly decreased (dark blue).

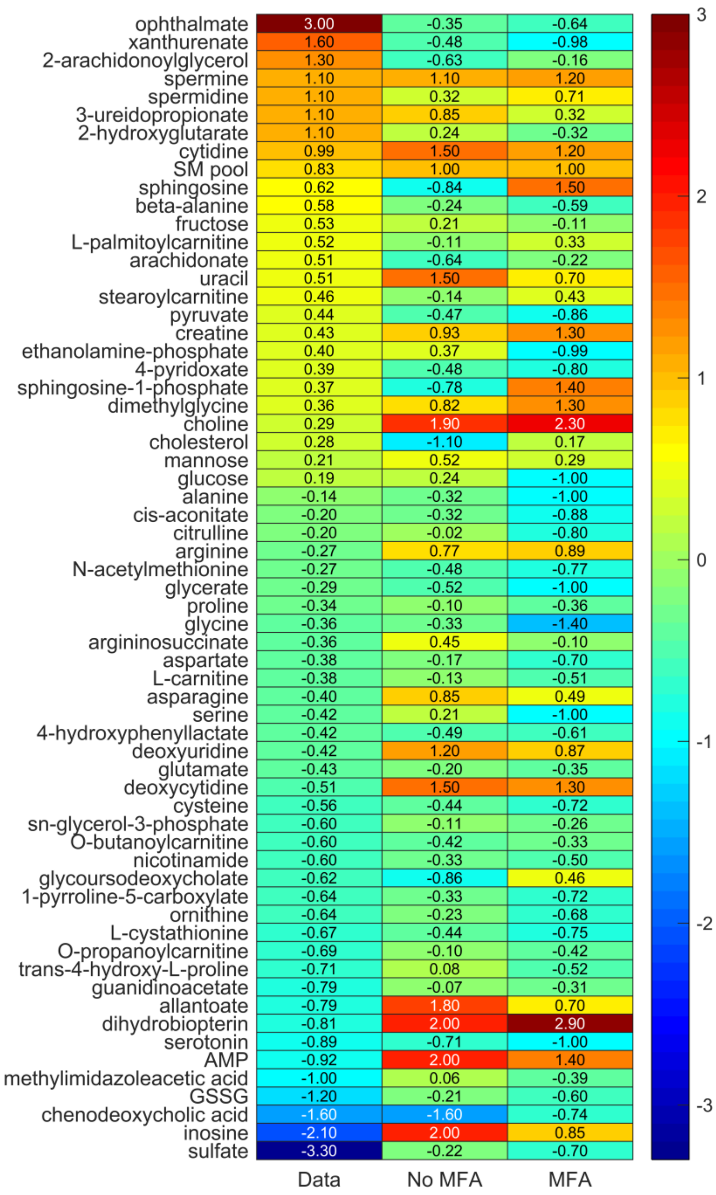

Supplementary Figure S2: Heat map of TIMBR production scores compared to metabolic profiling data obtained 10 h after APAP treatment under fasting conditions. *iRno* model predictions calculated under two integration conditions were compared against $\log_2$ fold changes of metabolites that significantly (FDR < 0.14) changed in the global metabolic profiling data (Data). In one condition, only gene expression changes were used (no MFA), whereas in the other both gene expression changes and MFA data were used as constraints (MFA). The numbers in the heat map show the $\log_2$ fold changes of the metabolic profiling data (left column) and TIMBR production scores under the no MFA (center column) and MFA (right column) conditions. The color scheme on the far right shows the degree of change in the level of a plasma metabolite, from highly increased (dark red) to highly decreased (dark blue).
